# Supplementary material for: Transition towards plate tectonics tracked in the metamorphic signature of Neoarchean synmagmatic transpression
Source: Nat Commun. 2025 Nov 27;16:10632. doi: 10.1038/s41467-025-65622-1 (PMC12660674; doi:10.1038/s41467-025-65622-1)
Supplement: Supplementary file 1 — Supplementary Information [file 41467_2025_65622_MOESM1_ESM.pdf]

Supplementary Material

i) Supplementary Text;

ii) Figures S1–S5;

iii) Supplementary Data 1;

iv) Supplementary Data 2

We selected three samples from the Youanmi Terrane (YT) and one sample from the Eastern Goldfield Superterrane (EGST) for detailed metamorphic analysis. Sample 199687 is a garnet-bearing amphibolite from the YT, which is representative of greenstone slivers that reached migmatitic conditions during the emplacement of the c. 2730 Ma Yarraquin pluton[1]. Sample 198197 from the YT is a porphyroblastic pelitic gneiss, representative of the synkinematic contact aureole of the c. 2680–2660 Ma Cundimurra pluton, which was emplaced along the Cundimurra shear zone[2]. Sample 198113 is a mylonitic granitic gneiss from the margin of the Cundimurra pluton, interpreted to reflect solid-state shearing during pluton cooling[3]. In the EGST, sample 240169 is a porphyroblastic pelitic to psammitic schist, which is representative of the synkinematic contact aureole of the 2680–2665 Ma Ballard pluton, which was emplaced along the Ballard shear zone[4].

Care was taken to ensure that the thin sections and the sample volume selected for whole-rock chemistry were similar in terms of featuring the same minerals in approximately the same abundances, to minimize any potential compositional differences.

1. Petrography

1.1 Sample 199687 (Migmatitic amphibolite – Yarraquin Pluton)

This sample contains hornblende, plagioclase, quartz, garnet and ilmenite. The gneissic foliation is defined by elongate hornblende and aggregates of quartz and plagioclase (Fig. S1a and b). Garnet grains of variable size (300–3000 µm) are distributed throughout the sample and vary from relatively inclusion free to poikiloblastic, including hornblende, plagioclase and quartz. Garnet typically occurs within mm- to cm-sized leucotonalitic pockets (Fig. S1a–c, see also Fig. 9i in[1]), interpreted to represent leucosomes. Garnet shows an internal foliation, defined by quartz, amphibole and plagioclase grains (Fig. S1d), which is subparallel to the

matrix foliation, indicating that garnet porphyroblasts grew on the existing foliation. However, the matrix foliation is also deflected along garnet porphyroblasts (Fig. S1d), demonstrating that garnet crystallised before the end of shearing in amphibolite.

Quartz is coarse grained (up to 1 mm in size) adjacent to garnet, and defines elongate ribbons, showing lobate boundaries against plagioclase and garnet (Fig. S1d), indicative of deformation at near-solidus temperatures[5]. Plagioclase (up to 750 μm in size) occurs throughout the sample. It is variably altered to fine-grained sericite and is freshest and coarsest in tonalitic domains. Ilmenite occurs as inclusions along the cleavage planes of hornblende, as well as throughout the matrix, and is locally included in garnet. Rare chlorite occurs as inclusions in garnet and in contact with hornblende, interpreted to be pseudomorphed hornblende.

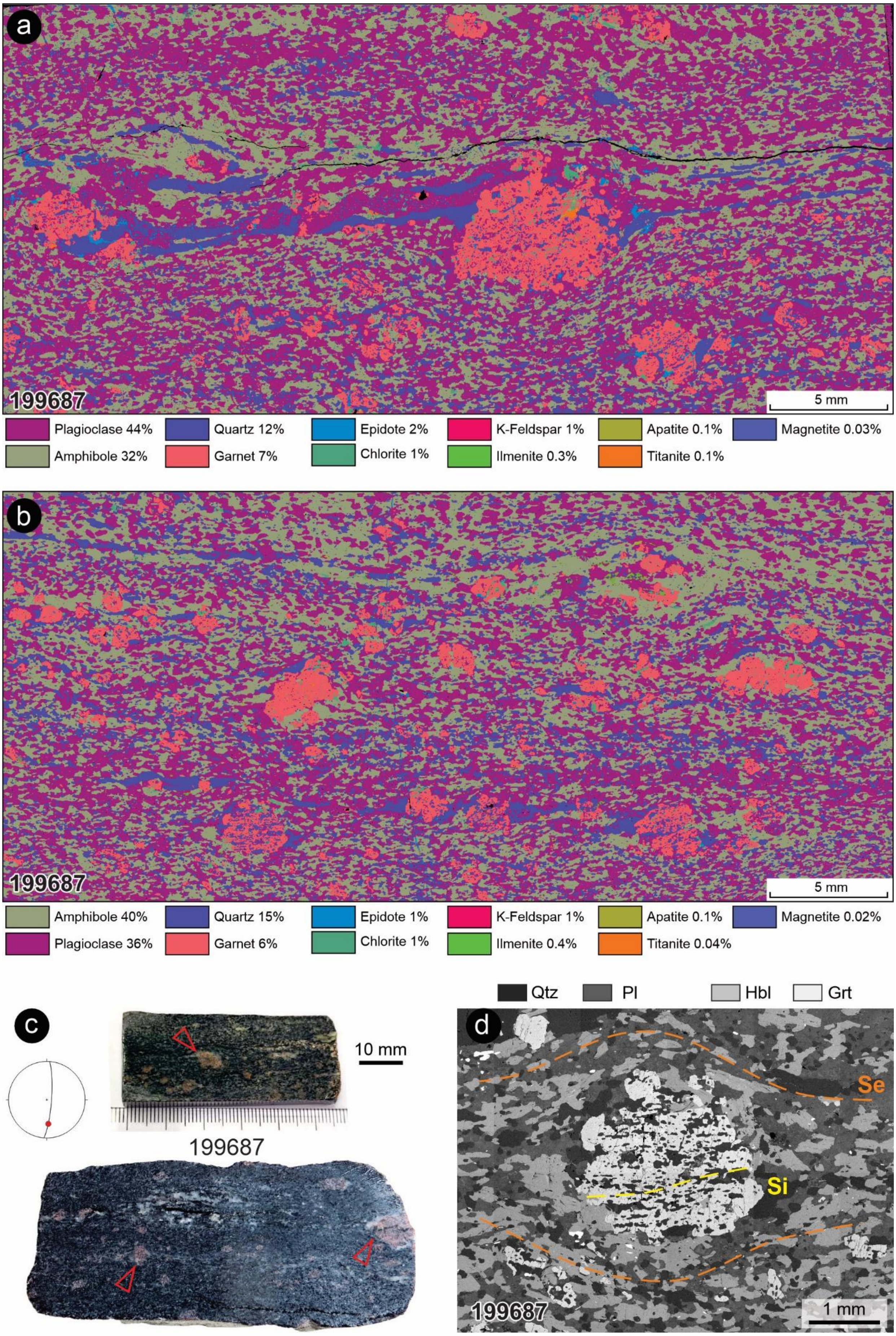
a
199687
5 mm
Plagioclase 44%
Amphibole 32%
Quartz 12%
Garnet 7%
Epidote 2%
Chlorite 1%
K-Feldspar 1%
Ilmenite 0.3%
Apatite 0.1%
Titanite 0.1%
Magnetite 0.03%
b
199687
5 mm
Amphibole 40%
Plagioclase 36%
Quartz 15%
Garnet 6%
Epidote 1%
Chlorite 1%
K-Feldspar 1%
Ilmenite 0.4%
Apatite 0.1%
Titanite 0.04%
Magnetite 0.02%
c
10 mm
199687
Qtz
Pl
Hbl
Grt
d
Se
Si
199687
1 mm

Figure S1a–d. Hand-sample-scale and chief microstructural features for the three YT samples. Lower hemisphere, equal-angle plots show the local orientation of foliation and lineation. (a) and (b) TESCAN Integrated Mineral Analyser (TIMA) image from two thin sections from sample 199687, highlighting its small-scale heterogeneity. Volume percent proportions of major rock-forming minerals are calculated by the TIMA software. (c) Hand sample and thin-section billet (top) for sample 199687. Red arrowheads point to representative examples of leucotonalitic pockets surrounding most garnet porphyroblasts. (d) Sample 199687, Backscattered-Electron (BSE) image, centred on a synkinematic garnet porphyroblast. The internal foliation (Si, dashed yellow line), mainly defined by elongated quartz grains, is subparallel to the matrix foliation (Se, dashed orange line), mainly defined by aligned quartz, plagioclase and hornblende aggregates. This microstructure indicates that garnet grew on an existing foliation. However, since Se wraps around the porphyroblast, its final stages of development must postdate garnet growth.

1.2 Sample 198197 (pelite – metamorphic aureole of Cundimurra Pluton)

This sample contains cm-sized andalusite porphyroblasts wrapped by a schistose fabric predominantly comprised of muscovite, ilmenite, plagioclase and quartz (Fig. S1e). Zibra et al.[2] (their locality C1) provided a detailed microstructural characterization of this sample, together with its mesoscale structural context. Andalusite grains are poikiloblastic and contain inclusions of oriented ilmenite and quartz, as well as porphyroblastic staurolite and plagioclase. The andalusite poikiloblasts have ragged edges and commonly contain muscovite-filled fractures that parallel the internal foliation. Inclusions within andalusite are aligned to define a sigmoidal internal foliation, which is continuous with the external, matrix foliation (Fig. S1e, f; see also Fig. 11c in Zibra et al.[2]). Staurolite is only found included in andalusite (Fig. S1e); it is typically up to 250 µm in size, itself containing inclusions of ilmenite and quartz that define an internal foliation, at a low to moderate angle from the internal foliation in the enclosing andalusite (Fig. S1g). Plagioclase porphyroblasts (typically 200–500 µm in size occur within andalusite grains and the matrix. As for andalusite, plagioclase contains a sigmoidal internal foliation (defined by muscovite flakes and ilmenite needles) continuous with the external, matrix foliation (Fig. S1h), demonstrating its synkinematic nature. Muscovite occurs within three distinct microstructural settings, with variable grain size. Fine-grained, poorly oriented muscovite typically occurs directly adjacent to the margin of andalusite porphyroblasts (1 in Fig. S1i), and likely results from replacement of andalusite. Coarser-grained, strongly aligned muscovite defines the main

metamorphic foliation at the hand-sample to thin-section scale (2, Fig. S1i, see also Fig. S1e), but it also occurs along a spaced and discontinuous crenulation cleavage at an angle to the main foliation (3, Fig. S1i), where it is associated with euhedral chloritoid porphyroblasts, which are up to 500 µm in size (Fig. S1i; see also Fig. 11e in Zibra et al.[2]).

Small, anhedral flakes of chlorite (< 200 µm), in places containing oriented ilmenite grains, occur in localised domains associated with albite, muscovite and extremely fine-grained paragonite. The latter, intergrown with fine-grained muscovite, occurs at the margins of plagioclase and andalusite porphyroblasts, and in domains that contain chlorite ± chloritoid.

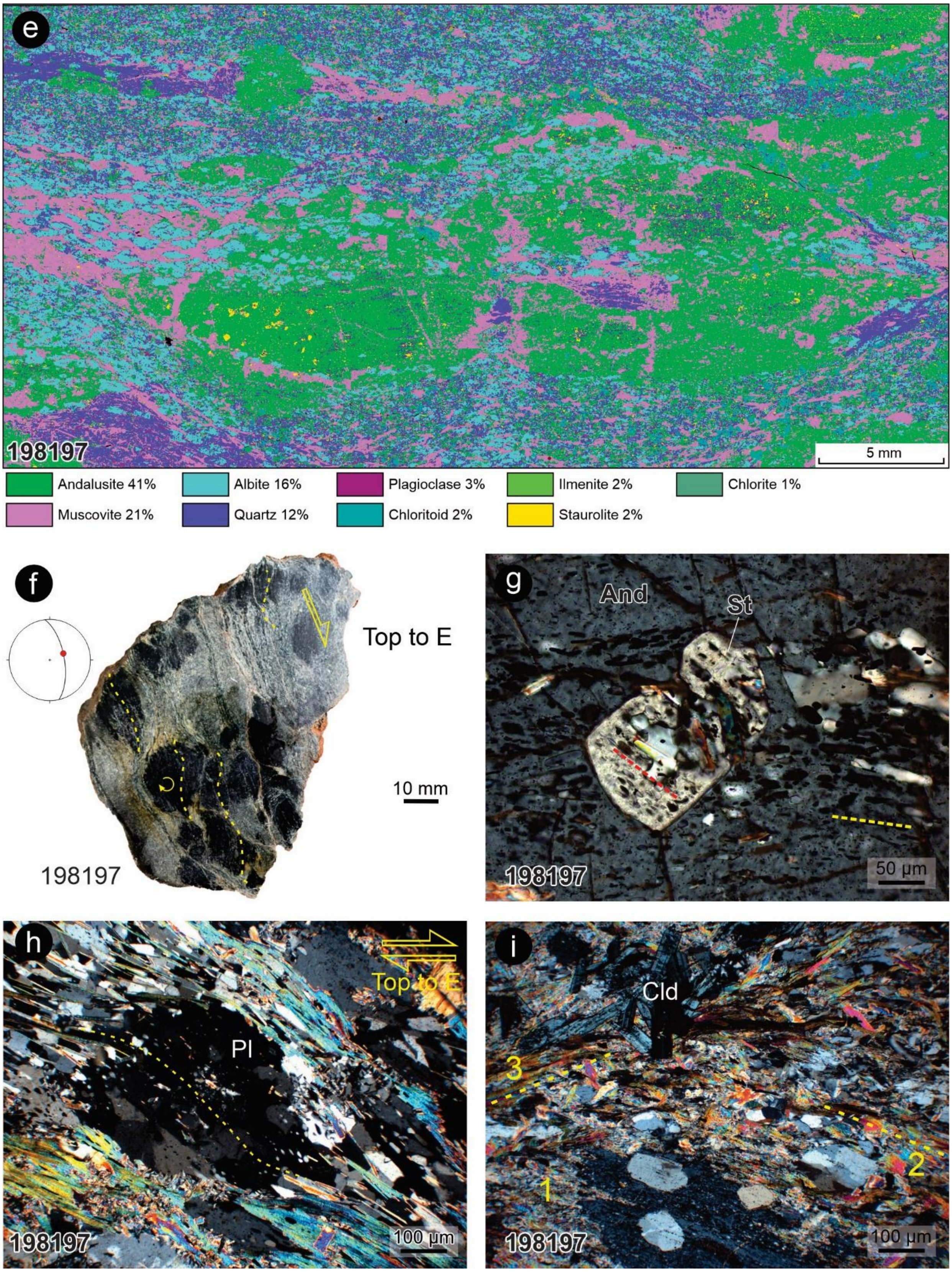
e
198197
5 mm
Andalusite 41%
Albite 16%
Plagioclase 3%
Ilmenite 2%
Chlorite 1%
Muscovite 21%
Quartz 12%
Chloritoid 2%
Staurolite 2%
f
Top to E
10 mm
198197
g
And
St
198197
50 µm
h
Pl
Top to E
198197
100 µm
i
Cld
3
2
1
198197
100 µm

Figure S1e–i. (e) TIMA image of the whole thin section from sample 198197. Volume percent proportions of major rock-forming minerals are calculated by the TIMA software. (f) Metapelite sample 198197 showing its true orientation in space (looking North). Cm-sized andalusite porphyroblasts show a sigmoidal internal foliation, which is continuous with the matrix foliation, and whose geometry implies up to 45° clockwise rotation during porphyroblast growth, in agreement with the overall top-to-NE shear sense[2]. g–i: microstructures from sample 198197. (g) Staurolite porphyroblast within andalusite. Both porphyroblasts show an internal foliation defined by aligned ilmenite and quartz grains, but the two foliations are discordant with each other. Crossed polars are used for all the micrographs presented here, unless indicated. (h) Plagioclase porphyroblast with sigmoidal internal foliation (dashed line) defined by muscovite, which is continuous with the matrix foliation, indicating about 30° rotation during porphyroblast growth. (i) Muscovite occurring in three distinct structural positions (1–3); rimming andalusite (1), defining the main foliation in the schist (2), and aligned along the chloritoid-bearing crenulation cleavage (3).

1.3 Sample 198113 (mylonitic granite – Cundimurra Pluton)

This sample is a medium-grained, protomylonitic to mylonitic biotite-bearing granite, containing plagioclase and K-feldspar porphyroclasts (former phenocrysts of magmatic origin) that are up to 10 mm in size (Fig. S1j). Mylonitization mainly took place within the quartz- and biotite-rich matrix in between more rigid feldspar porphyroclasts, which typically developed quartz-filled microfractures (Fig. S1j and k). In the matrix, the synkinematic metamorphic assemblage includes fine-grained aggregates (typically 50–250 µm in size, Fig. S1l) of quartz, biotite, muscovite, K-feldspar, albite, epidote and titanite. Here, quartz occurs as aggregates of polygonal grains 50–100 µm in size, Fig. S1l). Additional microstructural features of these synkinematic aggregates are shown in section 4.

Outside these mm-thick high-strain zones, primary biotite of magmatic origin is less strained and typically up to 1 mm in size (arrowhead in Fig. S1l), whereas quartz occur as anhedral grains up to several mm in size (Fig. S1j).

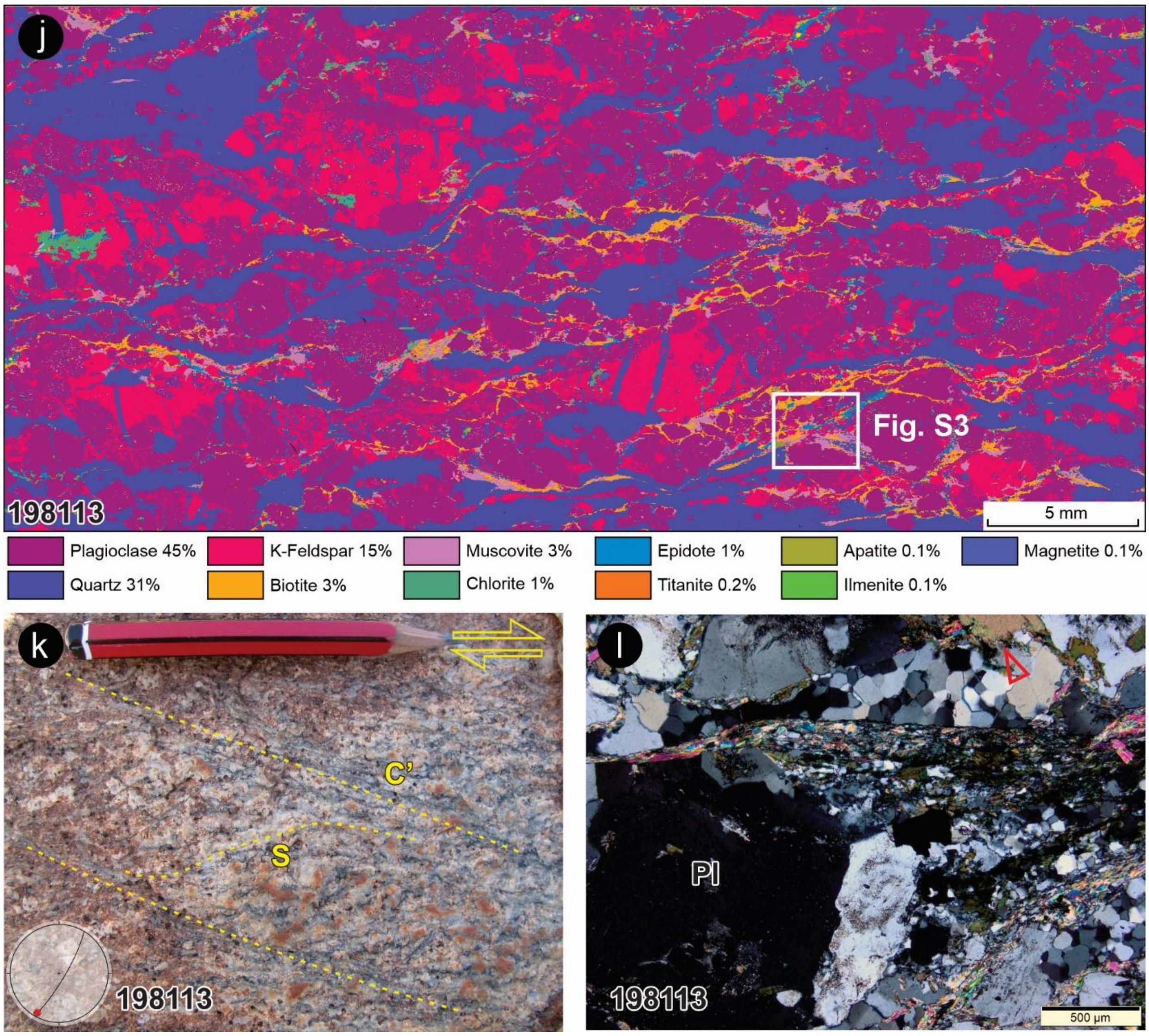

Figure S1j–l. (j) TIMA image of the whole thin section from sample 198113. Volume percent proportions of major rock-forming minerals are calculated by the TIMA software. (k) Outcrop-scale appearance of mylonitic gneiss sample 198113, showing C′ shear bands deflecting the gneissic foliation (S). (l) Typical microstructure from the sample shown in g, showing narrow high-strain zones containing the synkinematic recrystallized aggregate, here developed as tails around plagioclase porphyroclasts. Arrowhead indicates a large biotite flakes of magmatic origin, which is texturally distinct from the finer-grained biotite occurring within the recrystallised aggregate.

1.4 Sample 240169 (garnet–andalusite–staurolite–bearing cordierite pelitic schist – Ballard shear zone)

Sample 240169 is a foliated, fine- to medium-grained pelitic schist (Fig. S1m), containing 56% quartz, 17% plagioclase, 12% biotite, 8% cordierite, 2% garnet, 2% andalusite, 2% chlorite, <1%

staurolite, <0.5% ilmenite and accessory tourmaline, apatite, zircon, and monazite (supplementary data 1, Table S1). The schist displays a well-developed foliation defined by the preferential alignment of the main phases (Fig. S1m), with the biotite foliation wrapping around garnet and andalusite porphyroblasts. An in-depth microstructural characterization of this sample, and local mesoscale structural context may be found in Zibra et al.[4]. Quartz occurs as subhedral grains (typically 0.5–1 mm long) with lobate boundaries. The most quartz-rich regions are devoid of biotite and contain subhedral garnet and/or garnet intergrown with andalusite and staurolite (Fig. S1m, Fig. S1n and o). Plagioclase is fine-grained (< 1.5 mm long), subhedral and mainly occurs intergrown with biotite and in local regions devoid of coarse-grained cordierite (Fig. S1n). Biotite is fine-grained (up to 1mm), has stubby to acicular habit and defines the tectonic foliation (Fig. S1n).

Garnet is commonly anhedral and elongate in the fabric, with inclusions of staurolite, andalusite, plagioclase, ilmenite and quartz, that define an internal foliation (Si) subparallel to the matrix foliation (Se). Nevertheless, Se is also deflected along garnet porphyroblasts (Fig. S1o and p), indicating that garnet developed during the latest stages of fabric development. Staurolite is medium-grained (up to 0.6 mm in length), subhedral to prismatic and is commonly in direct contact with, or possibly included in, cordierite. Staurolite shows similar microstructures and Si-Se relationships as garnet (Fig. S1q), and can be therefore interpreted as late-kinematic, and coeval with garnet.

Andalusite may occur as garnet inclusions, with the latter developing thin, necklace-like coronae (Fig. S1r). Coarse-grained (about 100 µm to 1.5 mm), poikiloblastic andalusite exhibits fine-grained inclusions of plagioclase, biotite, apatite, ilmenite, quartz, and rare zircon. Andalusite is commonly mantled by a fringe of chlorite and muscovite. Direct contact between andalusite and garnet (Fig. S1r and s) and between andalusite and staurolite and cordierite, is locally observed. Cordierite is commonly subhedral, coarse-grained (up to 1.5 mm in length) and contains stubby, fine-grained inclusions of apatite, quartz, ilmenite, and biotite, commonly oriented with the main fabric (Fig. S1t). Staurolite and cordierite are commonly in direct contact.

Chlorite (< 1 mm long) occurs as rare grains within the matrix, and predominantly as symplectitic intergrowths (with sericite, plagioclase, and quartz) that mantle andalusite grains.

Rare ilmenite, tourmaline, apatite, and zircon occurs throughout the sample as thin to stubby, sub-angular grains that are commonly oriented with the main fabric.

Inclusions of staurolite and andalusite in garnet suggest that some garnet growth post-dates some staurolite and andalusite growth, and that these three minerals are in textural equilibrium. Staurolite, garnet and cordierite occur in direct contact (Fig. S1n and t), suggesting textural equilibrium. The simplest interpretation is that all main porphyroblastic minerals were in equilibrium at the metamorphic peak. Based on the Occam's razor approach, the interpreted subsolidus peak metamorphic assemblage is quartz–plagioclase–biotite–cordierite–garnet–staurolite–andalusite–ilmenite. Interpreted post-peak metamorphic to retrogressive mineralogy is limited to chlorite–sericite–plagioclase–quartz symplectites on andalusite. Since the main metamorphic phases are interpreted to have formed within an aureole during synkinematic granite emplacement along the transpressional Ballard shear zone, although microstructure may vary in detail as a function of strain partitioning between pure and simple shear components (Zibra et al., 2020).

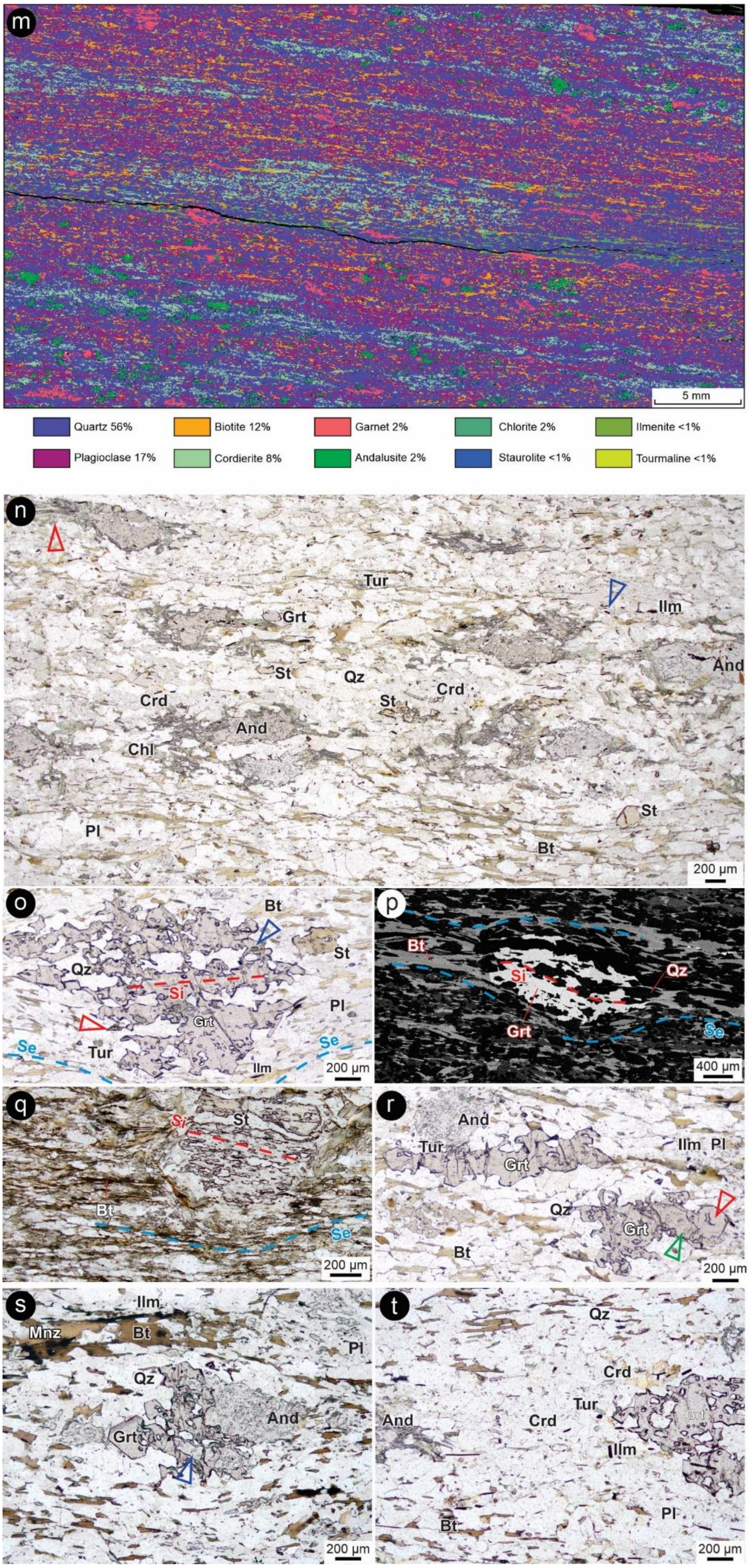
m
5 mm
Quartz 56%
Biotite 12%
Garnet 2%
Chlorite 2%
Ilmenite <1%
Plagioclase 17%
Cordierite 8%
Andalusite 2%
Staurolite <1%
Tourmaline <1%
n
Tur
Grt
Ilm
And
St
Qz
Crd
Chl
Pl
Bt
200 µm
o
Si
Se
200 µm
p
400 µm
q
r
s
Mnz
t

Figure S1m–t. (m) TIMA image of the whole thin section from sample 240169. Volume percent proportions of major rock-forming minerals are calculated by the TIMA software. (n)–(t): Photomicrographs from sample 240169. (n) Well-defined foliation and compositional layering marked by cordierite–quartz ± chlorite ± biotite interleaved with andalusite–staurolite ± biotite ± chlorite-rich layers, together with garnet-rich domains devoid of biotite, and often encased by quartz. Euhedral poikiloblasts of medium-grained staurolite and garnet, together with subhedral andalusite and cordierite pepper this layering. Blue arrowhead points to euhedral fine-grained ilmenite needles. Red arrowhead points to matted chlorite (secondary symplectite) on andalusite. (o) Garnet poikiloblasts including the matrix foliation (subhorizontal). Garnet contains inclusions of quartz, ilmenite, and tourmaline (blue arrowhead). Locally, corroded, fine-grained staurolite (red arrowhead) also present. (p) BSE image centred on a synkinematic garnet porphyroblast. A sigmoidal internal foliation (Si, red dashed line), mainly defined by elongated quartz grains, is subparallel to the matrix foliation (Se), mainly defined by aligned quartz and biotite aggregates. This microstructure indicates that garnet grew on an existing foliation. However, since the matrix foliation wraps around the porphyroblast, its final stages of development must postdate garnet growth. (q) Synkinematic staurolite porphyroblast, showing an internal foliation (Si), mainly defined by elongated quartz grains. Similarly to what observed for garnet, the matrix foliation (Se) is largely parallel to the internal one, but is also partially deflected around the porphyroblast, indicating its synkinematic nature. (r) Delicate necklacing of poikiloblastic andalusite by garnet (green arrowhead). Fringe garnet is subhedral, displaying delicate vermicular protrusions and embayment. Curvilinear to corroded, fine-grained inclusion of staurolite (red arrowheads) also present. (s) Detail of garnet poikiloblasts displaying euhedral margins against quartz and a delicate necklacing of andalusite (blue arrowhead). Note the fine-grained poikiloblastic nature of andalusite. Monazite radiation halos occur within biotite grains to the top left. (t) Detail of adjacent garnet and cordierite poikiloblasts, both containing inclusions of quartz, tourmaline, and ilmenite. Note that garnet–cordierite domains are relatively biotite poor.

2. Whole-rock major and trace element analyses

Whole rock data are provided in supplementary data 1, Table S2.

3. Mineral chemistry

Representative chemical analyses of minerals are provided in supplementary data 1, Table S3.

3.1 Results

3.2

Sample 199687 (migmatitic amphibolite – Yarraquin pluton)

Garnet is weakly zoned (Fig. S2a) and almandine rich, with $Fe^{2+}/(Fe^{2+} + Mg + Mn + Ca)$ compositions higher at the rim (0.59) and lower (0.56–0.57) in the core. Pyrope contents $[Mg/(Fe^{2+} + Mg + Mn + Ca)]$ are dominantly 0.17–0.19 in the core and 0.14–0.16 at the rim. Spessartine contents $[Mn/(Fe^{2+} + Mg + Mn + Ca)]$ show a small zone of enrichment in the core (0.09), decreasing to 0.07 in the rims, before increasing to 0.12 at the very edge of the grain. Grossular contents $[Ca/(Fe^{2+} + Mg + Mn + Ca)]$ are similar across the garnet, with values of 0.16 in the core and 0.15 in the rims. *Hornblende* is magnesio-hornblende [6] with X_{Fe} of 0.37–0.43, TiO_2 contents of 0.66–1.12 wt%, , MnO contents of 0.39–0.54 wt% and low F contents of 0–0.03 ions pfu (23 oxygen basis). *Plagioclase* has an intermediate composition with X_{Ab} = 0.57–0.61 and negligible K contents. *Ilmenite* has MnO contents of 4.19–6.98 wt%.

Sample 198197 (pelite – Aureole of Cundimurra Pluton)

Staurolite has X_{Fe} $[Fe^{2+}/(Fe^{2+} + Mg)]$ of 0.90–0.92, ZnO contents of 0.03–2.43 wt% and Cr2O3 contents of 0.45–0.58 wt%. *Plagioclase* is albitic with X_{Ab} [Na/(Na + Ca+ K)] of 0.97–0.98. *White mica* has two distinct compositions, one with a muscovite content of 0.62–0.72 and another with a paragonite content of 0.71–0.94 and a margarite component of 0.03–0.04. *Chloritoid* has X_{Fe} of 0.91–0.93 and MnO contents of 0.17–0.25 wt%. *Chlorite* has X_{Fe} of 0.69–0.70 and MnO contents of 0.06–0.1 wt%. *Ilmenite* has MnO contents of 0.02–0.49 wt% and *andalusite* contains 0.39–0.41 wt% FeO.

Sample 198113 (mylonitic granite – Cundimurra Pluton)

Plagioclase porphyroblasts are dominantly albitic (X_{Ab} = 0.81–0.88) with sanadine contents [X_{san} = K/(Na + Ca+ K) of 0–0.01. *K-feldspar* is sandine rich (X_{san} = 0.96–0.97) with minor X_{Ab} of 0.03–

0.04. *Biotite* has X_{Fe} of 0.54–0.58 with MnO contents of 0.32–0.39 wt%. *Muscovite* has paragonite contents of 0.03–0.4 and elevated FeO and MgO contents of 4.61–5.31 and 1.23–1.51 wt%. *Chlorite* has X_{Fe} of 0.57–0.58 and MnO contents of 0.61–0.71 wt%. *Epidote* with two compositions can be identified on the MLA map; one with lower TiO_2 (0.05-0.24 wt%), and total measured FeO (11.00–14.74 wt%) and higher Al contents of 1.97–2.30 cations per formula unit (cpfu, 12.5 oxygen basis), and a second with higher TiO_2 of 0.99 wt% and FeO of 19.29 wt% and lower Al contents of 0.96 cpfu.

Sample 240169 (pelitic to psammitic schist – Ballard Shear Zone)

Garnet is weakly zoned (Fig. S2b) and almandine rich, with higher almandine contents at the rim (0.82) than in the core (0.74–0.75). Pyrope contents [$Mg/(Fe^{2+} + Mg + Mn + Ca)$] are low at the rim (0.05) and higher in the core (0.07 – 0.08). Spessartine contents [$Mn/(Fe^{2+} + Mg + Mn + Ca)$] are lower at the rim (down to 0.10) and higher in the core (0.16 – 017). Grossular contents are the same for rim and core (0.02). *Biotite* is Fe-rich (X_{Fe} = 0.65–0.67) and has TiO_2 contents of1.23 – 1.62 wt%. Fluorine and chlorine ions in biotite are 0.07 – 0.07 and 0.00 ions pfu, respectively (11 oxygen basis). *Muscovite* has paragonite content of 0.12 – 0.14. *Magnetite* is pure and *sillimanite* contains 0.78 – 1.66 wt% FeO.

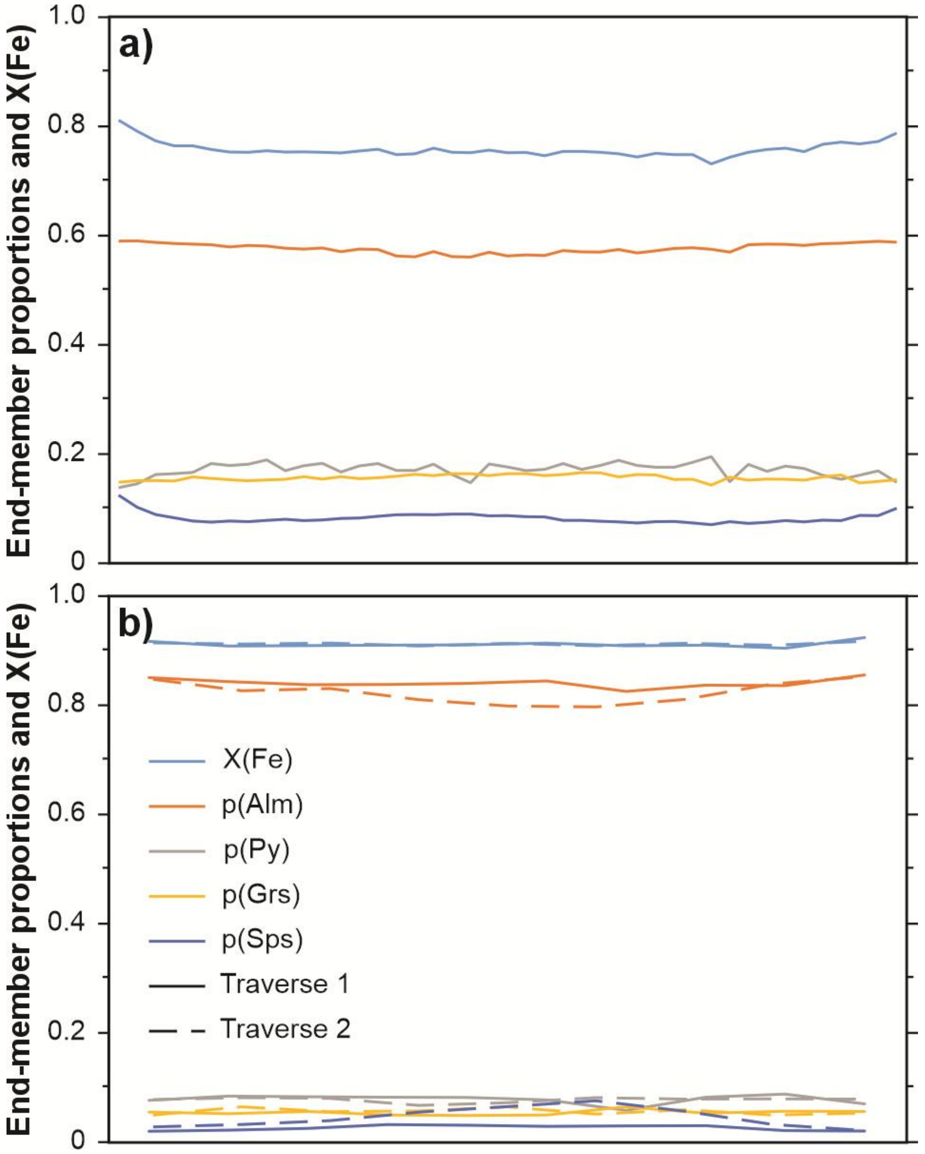

Figure S2. Traverses across representative garnet porphyroblasts from samples 199687 (a) and 240169 (b), obtained by electron probe microanalyzer (EPMA). X(Fe) = $Fe^{2+}/(Fe^{2+} + Mg)$; p(Alm) = $Fe^{2+}/(Fe^{2+} + Mg + Ca + Mn)$; p(Py) = $Mg/(Fe^{2+} + Mg + Ca + Mn)$; p(Grs) = $Ca/(Fe^{2+} + Mg + Ca + Mn)$; p(Sps) = $Mn/(Fe^{2+} + Mg + Ca + Mn)$. Full compositional data provided in supplementary data 1, Table S3.

4. Phase equilibria modelling

Models contoured for selected isopleths are presented in Supplementary Figures S4 and S5.

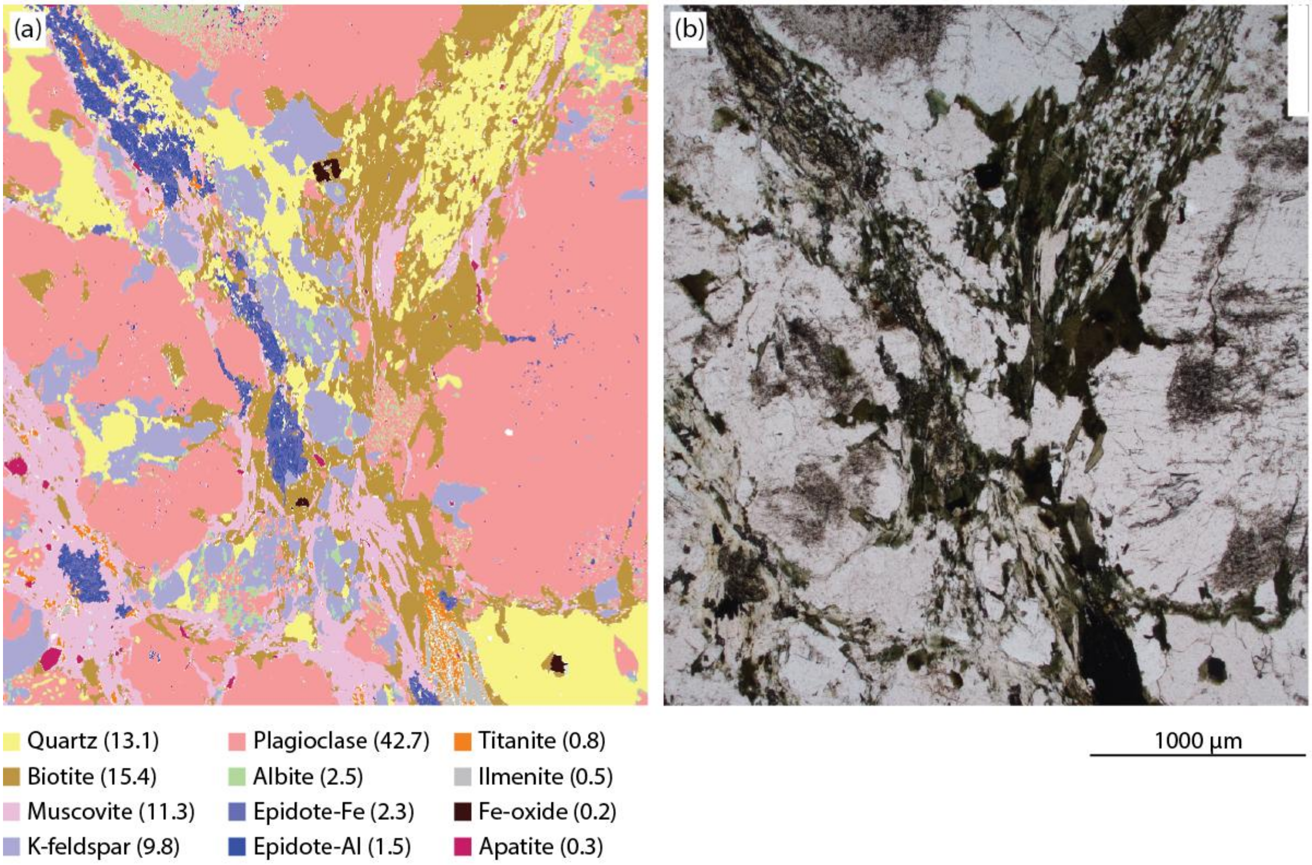

Figure S3: Images showing area (~3 x 3 mm) used for calculating the domainal bulk composition for sample 198113. (a) Mineral Liberation Analysis map with wt% values of each mineral provided below. (b) Plane polarised light image of the same area.

Table S4: Proportions and representative mineral compositions used for the calculation of the bulk composition.

4.1 Pressure–temperature modelling results.

Sample 199687 (migmatitic amphibolite – Yarraquin Pluton)

The peak assemblage in this sample is garnet + hornblende + plagioclase + ilmenite + quartz + melt. This assemblage occurs in a large *P–T* field between 4.5–10 kbar and 730–825 °C. The absence of orthopyroxene and rutile constrain the minimum and maximum pressures, respectively. The tonalitic domains that wrap garnet are interpreted to reflect the former presence of melt[1], suggesting temperatures in excess of 730 °C. The absence of augite provides an upper temperature constraint of 825 °C. We refined peak *P–T* conditions by using modal proportion estimates derived from TIMA maps of two thin sections, where vol% from the TIMA maps is approximately equivalent to mol% in the *P–T* models. The modal proportion (Fig. S4) of garnet (~ 6–7 vol.%) and hornblende (~ 32–40 vol.%) suggest *P–T* conditions of 5–7.5 kbar and 750–810 °C. The observed proportions of plagioclase (36–44 vol.%) and quartz (~12–15 vol.%) are consistent with this *P–T* range. The absence of Mn-bearing a–x models for hornblende, augite and tonalite melt is a limitation of the modelling that may result in slightly increased stability of garnet. However, the MnO content of hornblende in the sample is minor (<0.5 wt%) compared to the MnO contents of garnet and ilmenite (3.1–5.6 wt% and 4.2–6.9 wt%, respectively) and is therefore not interpreted to significantly change the peak *P–T* conditions.

Sample 198197 (pelite – metamorphic aureole of Cundimurra Pluton)

The peak assemblage in this sample is plagioclase + muscovite + andalusite + ilmenite + quartz. Staurolite occurs as inclusions within andalusite porphyroblasts and so is interpreted to be a prograde mineral rather than part of the peak assemblage. The field that best corresponds to the peak assemblage occurs at pressures below 3.7 kbar and temperatures of 480–630 °C. This field is predicted to contain ~4 mol% biotite, which is not present in the rock. The presence of biotite in the peak field is likely to reflect the presence of small amounts of MgO in the bulk composition that is contained in chlorite and chloritoid (below) but cannot be incorporated in significant amounts into any of the peak minerals. However, the presence of andalusite rather than sillimanite provides a robust upper pressure constraint that is not affected by the prediction of minor amounts of biotite in the peak field. Contouring the peak field for the modal proportion of minerals does not constrain conditions further.

This sample contains post-peak chlorite, chloritoid and paragonite, and fine-grained muscovite that may also be retrograde (domain 1 in Fig. S1i). There is no evidence for secondary

staurolite. Chloritoid is oriented along a spaced crenulation cleavage, together with muscovite (domain 3 in Fig. S1d). It is therefore likely to reflect recrystallisation of some domains of the rock during a post-peak deformation event. It is possible that the chloritoid–chlorite–paragonite assemblage reflects a retrograde mineral assemblage that grew at temperatures below 500 °C. However, as this retrograde assemblage is domainal and may be related to fluid ingress along cleavage planes, it is not possible to obtain robust *P*–*T* constraints using the whole-rock bulk composition.

Sample 198113 (mylonitic granite – Cundimurra pluton)

The mineral assemblage in the recrystallised domains is plagioclase + epidote + K-feldspar + biotite + muscovite + albite + titanite. This assemblage occurs in a relatively narrow field that extends from the edge of the diagram at 1.4 kbar and 450 °C to 5.8 kbar and 545 °C. The absence of garnet in the sample provides an upper pressure constraint, whereas the presence of epidote and titanite provide an upper temperature constraint. Modal proportion and compositional contours are parallel to the boundaries of this field and therefore do not constrain *P*–*T* conditions further. Assuming that pressures in this sample do not exceed the peak pressures in the contact aureole, the *P*–*T* conditions of shearing were less than 3.7 kbar and 495 ± 10 °C. These *P*–*T* conditions of the domanial assemblage in this sample correspond to the chlorite–chloritoid–muscovite–paragonite field in sample 198197.

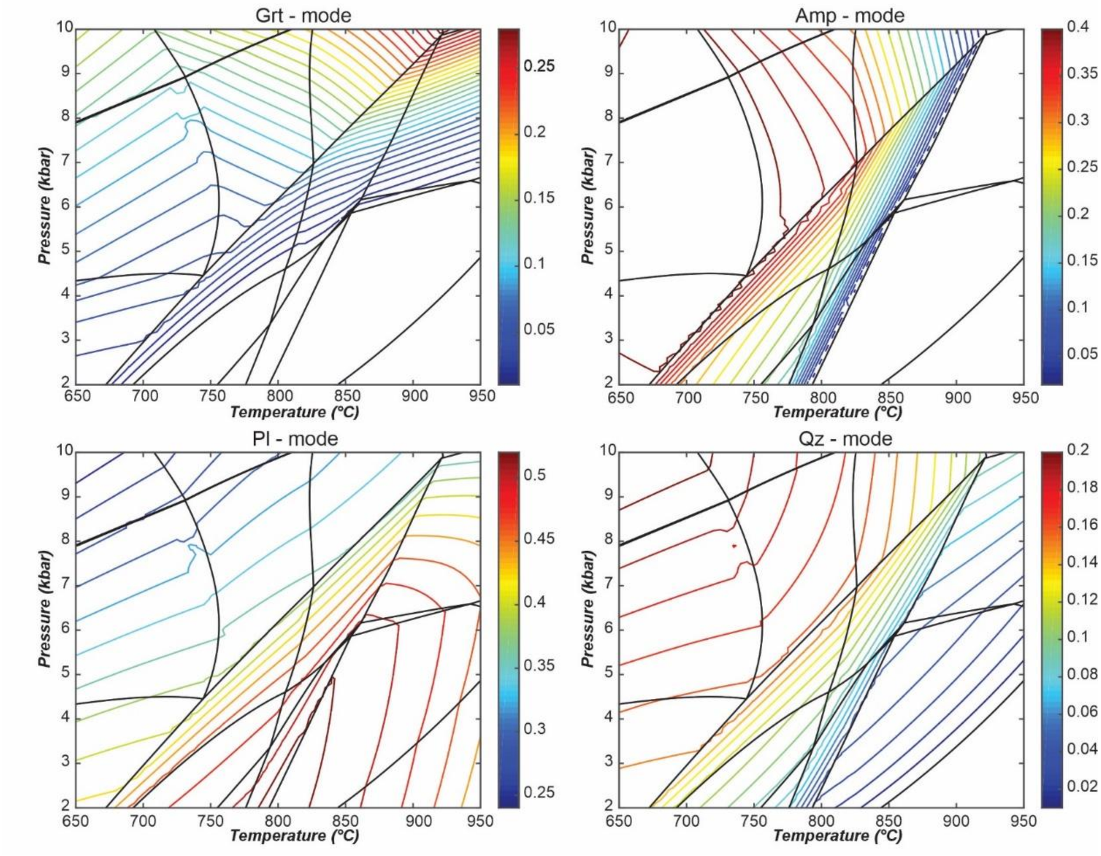

Figure S4: Calculated volume percent modal proportion data for sample 199687.

Sample 240169 (pelitic to psammitic schist –Ballard shear zone)

The *P*–*T* pseudosection for sample 240169 was calculated over a pressure range of 1–4 kbar and temperature range of 450–610 °C (Fig. 2). Quartz, biotite, plagioclase, ilmenite, and magnetite are stable everywhere across the modelled *P*–*T* range. Garnet is not stable in the high-temperature–low-pressure part of the modelled *P*–*T* range, below about 1.9 kbar, 540 °C and 2.6 kbar, 610 °C. Cordierite is stable at pressures below about 2 kbar, 460 °C and 4 kbar, 590 °C. Andalusite is stable below at pressures below about 1.4 kbar, 500 °C and 3.5 kbar, 600 °C. Staurolite is stable at pressures above about 1 kbar, 475 °C and 3.6 kbar, 610 °C. Sillimanite is stable within the high temperature–high pressure corner, above the aluminosilicate polymorph boundary superseding andalusite. K-feldspar is stable at the extreme low pressure–high temperature corner. Free fluid is stable within a low pressure–high temperature sector of the modelled *P*–*T* space. Chlorite is stable to lower temperatures than about 530 °C at 1.6 kbar and 600 °C at 4 kbar.

The simplest interpreted peak mineral assemblage of quartz–plagioclase–biotite–cordierite–garnet–andalusite–staurolite–ilmenite–magnetite and aqueous fluid (order of modal

abundance) is stable over the *P*–*T* range 1.7–3.5 kbar and 520–605 °C. The sample, and all schists within the studied region, show no evidence of partial melting[4], meaning that peak metamorphism did not reach the solidus. The absence of sillimanite from the sample limits the maximum pressure for metamorphism. The lower pressure constraint is provided by the presence of garnet. Retrogression of the sample is limited to late chlorite on andalusite, therefore there is no petrological information to constrain any *P*–*T* path information.

375

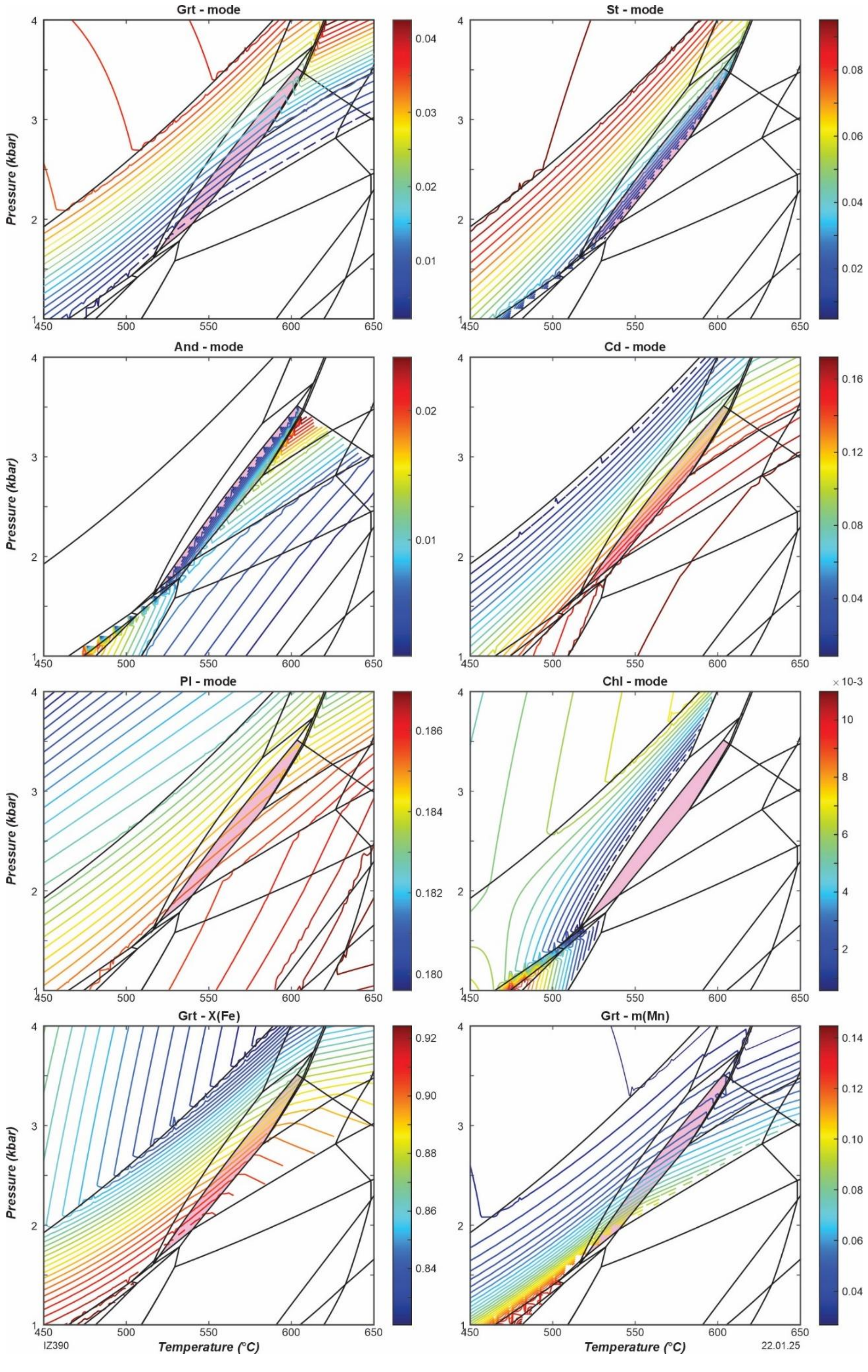
Grt - mode
St - mode
And - mode
Cd - mode
Pl - mode
Chl - mode
Grt - X(Fe)
Grt - m(Mn)
Pressure (kbar)
Temperature (°C)
IZ390
22.01.25

Figure S5: Calculated phase volume percent modal proportions (approximately equal to volume percent) for selected minerals and solid-solution compositional data for sample 240169. Labelled *P*–*T* pseudosection shown in Figure 4. See Appendices 1 and 3 for definitions of compositional variables. Growth of cordierite and locally andalusite at the partial expense of garnet and andalusite and near complete dissolution of staurolite occurred with an increase in temperature, as shown by the modal variances across the modelled *P*–*T* space. Inversely, the growth of chlorite supersedes cordierite down temperature, if a post-peak cooling path is evoked. Proxies for end-member compositions xFe=X(Fe) and Xm(Mn)=X(Sps) for garnet, which semi-quantitatively suggest a medium pressure stability for garnet within the peak assemblage field, but is unsubstantiated given the caveats given modelled compositional isopleths.

References

1. Zibra, I., Clos, F., Weinberg, R. F. & Peternell, M. The c. 2730 Ma onset of the Neoarchean Yilgarn Orogeny. *Tectonics* **36,** 1787–1813; 10.1002/2017TC004562 (2017).
2. Zibra, I., Smithies, R. H., Wingate, M. T. D. & Kirkland, C. L. Incremental pluton emplacement during inclined transpression. *Tectonophysics* **623,** 100–122; 10.1016/j.tecto.2014.03.020 (2014).
3. Zibra, I., White, J. C., Menegon, L., Dering, G. & Gessner, K. The ultimate fate of a synmagmatic shear zone: interplay between rupturing and ductile flow in a cooling granite pluton. *Journal of Structural Geology* **110,** 1–23; 10.1016/j.jsg.2018.02.001 (2018).
4. Zibra, I., Weinberg, R. F. & Peternell, M. Neoarchean synmagmatic crustal extrusion in the transpressional Yilgarn Orogen. *Tectonics* **39**; 10.1029/2019TC005947| (2020).
5. Gower, R. J. W. & Simpson, C. Phase boundary mobility in naturally deformed, high-grade quartzofeldspathic rocks: evidence for diffusional creep. *Journal of Structural Geology* **14,** 301–313; 10.1016/0191-8141(92)90088-E (1992).
6. Hawthorne, F. C. *et al.* Nomenclature of the amphibole supergroup. *American Mineralogist* **97,** 2031–2048; 10.2138/am.2012.4276 (2012).
