# Supplementary material for: Transition towards plate tectonics tracked in the metamorphic signature of Neoarchean synmagmatic transpression
Source: Nat Commun. 2025 Nov 27;16:10632. doi: 10.1038/s41467-025-65622-1 (PMC12660674; doi:10.1038/s41467-025-65622-1)
Supplement: Supplementary file 2 — Description of Additional Supplementary Information [file 41467_2025_65622_MOESM2_ESM.pdf]

Description of Additional Supplementary Information

Supplementary Data 1:

Table S1. Mineral modes for sample 240169: cordierite-garnet pelitic schist, Lawlers – mount Ida road.

Table S2. Whole-rock major and trace-element compositions of the samples studied in this work.

Table S3. EPMA mineral compositional data.

Table S4: Proportions and representative mineral compositions used for the calculation of the bulk composition for domainal sample 198113 .

Supplementary Data 2:

Modified white mica a-x file for use in THERMOCALC v3.4, provided by Tim Holland (2017).
